# Proceedings of the 16th Annual UT-KBRIN Bioinformatics Summit 2016: proceedings: Burns, TN, USA. April 21-23, 2017

**DOI:** 10.1186/s12919-017-0078-5

**Published:** 2017-10-09

**Authors:** L. Leon Dent, Sammed N. Mandape, Siddharth Pratap, Jianan Dong, Jamaine Davis, Jennifer A. Gaddy, Kofi Amoah, Steve Damo, Dana R. Marshall, Jacob Jones, Toni Brandt, Gilberto Diaz, Qingguo Wang, Todd Gary, Ashwini Yenamandra, Marina Z. Ghattas, Marwa Elrakaiby, Ramy K. Aziz, Hamdallah H. Zedan, Moamen Elmassry, Marwa ElRakaiby, Ramy K. Aziz, Mariam Lotfy, Moamen Elmassry, Jarrad Marcel, Rania A. Khattab, Maha M. Abdelfattah, Jack A. Gilbert, Ramy K. Aziz, Pouya Dini, Shavahn C. Loux, Kirsten E. Scoggin, Alejandro Esteller-Vico, Edward L. Squires, Mats H. T. Troedsson, Peter Daels, Barry A. Ball, Kalpani De Silva, Ernest Bailey, Joel C. Stephens, Theodore S. Kalbfleisch, Christine E. Dolin, Lauren G. Poole, Daniel W. Wilkey, Eric C. Rouchka, Gavin E. Arteel, Michelle T. Barati, Michael L. Merchant, Richard M. Higashi, Teresa W-M Fan, Hunter Moseley, Andrew N Lane

**Affiliations:** 1 Department of Surgery, Meharry Medical College, Nashville, TN 37208 USA; 2 Bioinformatics Core, Meharry Medical College, Nashville, TN 37208 USA; 3 Department of Biochemistry and Cancer Biology, Meharry Medical College, Nashville, TN 37208 USA; 4 Department of Medicine, Vanderbilt University Medical Center, Nashville, TN 37232 USA; 5 Department of Life and Physical Sciences, Fisk University, Nashville, TN 37208 USA; 6 Department of Pathology, Anatomy and Cell Biology, Meharry Medical College, Nashville, TN 37208 USA; 7 College of Computing and Technology, Lipscomb University, 1 University Park Drive, Nashville, TN 37204 USA; 8 Office of Research, Middle Tennessee State University, 1301 East Main St, Murfreesboro, TN 37132 USA; 9 Vanderbilt University Medical Center, Nashville, TN 37232 USA; 10 Department of Microbiology and Immunology, Faculty of Pharmacy, Cairo University, Cairo, Egypt; 11 Department of Biological Sciences, Texas Tech University, Lubbock, TX 79409 USA; 12 Department of Microbiology and Immunology, Faculty of Pharmacy, Cairo University, Cairo, Egypt; 13 Clinical Operations Department, Ray-Clinical Research Organization, Giza, Egypt; 14 Department of Biological Sciences, Texas Tech University, Lubbock, TX 79409 USA; 15 Genomic and Systems Biology, Bioscience Division, Argonne National Laboratory, Argonne, IL 60439 USA; 16 Department of Microbiology and Immunology, Faculty of Pharmacy, Cairo University, Cairo, Egypt; 17 Research Institute of Ophthalmology, Giza, Egypt; 18 Department of Surgery, University of Chicago, Chicago, IL 60637 USA; 19 Microbial Ecology Group, Argonne National Laboratory, Argonne, IL 60439 USA; 20 Department of Veterinary Science, University of Kentucky, Lexington, KY 40536 USA; 21 Department of Reproduction, Faculty of Veterinary Medicine, University of Gent, Merelbeke, Belgium; 22 Interdisciplinary Studies Program: Specialization in Bioinformatics, University of Louisville, Louisville, KY 40292 USA; 23 Department of Veterinary Science, University of Kentucky, Lexington, KY 40506 USA; 24 Genomics GPS, Guilford, CT 06437 USA; 25 Department of Biochemistry and Molecular Genetics, School of Medicine, University of Louisville, Louisville, KY 40292 USA; 26 Department of Pharmacology & Toxicology, University of Louisville, Louisville, KY 40202 USA; 27 Department of Medicine, University of Louisville, Louisville, KY 40202 USA; 28 Department of Computer Engineering & Computer Science, University of Louisville, Louisville, KY 40202 USA; 29 Center for Environmental and Systems Biochemistry, University of Kentucky, Lexington, KY 40536 USA; 30 Department of Toxicology and Cancer Biology, University of Kentucky, Lexington, KY 40536 USA; 31 Markey Cancer Center, University of Kentucky, Lexington, KY 40536 USA

## P1 Proteogenomic characterization of a clinical isolate of *Acinetobacter baumanii* from a case of fulminant sepsis: What does the data mean clinically?

### L Leon Dent^1^, Sammed N Mandape^2^, Siddharth Pratap^2^, Jianan Dong^2^, Jamaine Davis^3^, Jennifer A Gaddy^4^, Kofi Amoah^5^, Steve Damo^5^, Dana R Marshall^6^

#### ^1^Department of Surgery, Meharry Medical College, Nashville, TN 37208, USA; ^2^Bioinformatics Core, Meharry Medical College, Nashville, TN 37208, USA; ^3^Department of Biochemistry and Cancer Biology, Meharry Medical College, Nashville, TN 37208, USA; ^4^Department of Medicine, Vanderbilt University Medical Center, Nashville, TN 37232, USA; ^5^Department of Life and Physical Sciences, Fisk University, Nashville, TN 37208, USA; ^6^Department of Pathology, Anatomy and Cell Biology, Meharry Medical College, Nashville, TN 37208, USA

##### **Correspondence:** Dana R Marshall (dmarshall@mmc.edu)

**Background**

A middle-aged African American male with a history of HIV/AIDS was admitted to Nashville General Hospital at Meharry (NGHM), with severe diarrhea, nausea and vomiting. Upon admission, he was diagnosed with sepsis of unknown etiology. During his hospitalization of almost one month, he stabilized with supportive care but ultimately required ventilation for several days. Efforts to isolate microbes were unsuccessful until one week after mechanical ventilation, when multi-drug resistant *Acinetobacter baumanii* was isolated from sputum, BAL, blood and urine. Genomic and proteomic analyses were used to better understand the course of this infection with the goal of identifying potential diagnostic markers of virulence and therapy.

**Materials and methods**

Whole genome shotgun sequencing consisted of single-end 43 bp reads by Illumina GA-IIx and paired-end 250 bp reads (with 2 kb and 5 kb inserts) on the Roche 454 platform. For proteome analysis, the isolate was cultured in the presence and absence of antibiotics (levofloxacin, tobramycin, gentamycin, cefotaxime, and meropenem) and tandem liquid chromatography mass spectrometry (LC-MS/MS) was used to identify proteins expressed, and comparative protein expression, between the two culture conditions.

**Results**

**Fig. 1 (abstract P1).**
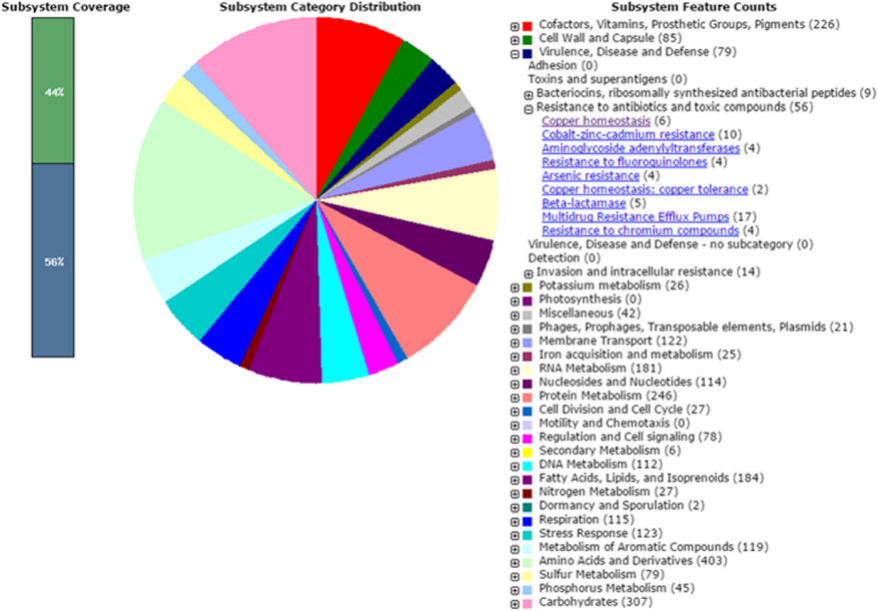
RAST server annotations for *A. baumanii* clinical isolate. Genes associated with virulence are highly represented.

A hybrid *de novo* assembling technique yielded a draft genome of 3,985,367 bp length, 148 contigs, and an N50 of 66,205 bp (GenBank accession - AZNQ00000000). The NCBI Prokaryotic Genome Automatic Annotation Pipeline predicted 4,064 genes; many of them without annotations or known functions. RAST server annotation reported 79 genes in the subsystem ‘Virulence, Disease, and Defense’ and 21 genes in the ‘Phages, Prophages, Transposable elements, Plasmids’ (Fig. 1). Proteome analysis identified greater than 400 proteins total. Comparative protein expression with and without antibiotics, showed 21 proteins differentially expressed (*p* ≤ 0.005) with the significantly elevanted presence of superoxide dismutase suggesting a strong resistance mechanism that is dangerously broad in scope.

**Conclusions**

MDR *A. baumanii* was previously identified as bacteria frequently isolated at NGHM [1]. The WHO lists it as a leading drug resistant pathogen in the hospital environment [2]. Data gathered previously [3], and in this study, clearly show a high representation of genes associated with virulence, disease and defense in this MDR clinical isolate. Proteins differentially expressed suggest an initial non-antibiotic specific mechanism of resistance. Diagnostics targeting these genes or proteins quite likely would have shortened the progression of infection in this patient. There is a critical need to identify mechanisms of virulence, persistence and antimicrobial resistance for *A. baumanii* and systems biology approaches substantially contribute to this knowledge database.

**Acknowledgements**

NIH grants MD007586 (RCMI) and MD007593 (MeTRC) from the National Institute on Minority Health and Health Disparities (NIMHD). The work presented herein was approved by the Meharry Medical College Institutional Review Board.

References

1. Dent LL, Marshall DR, Pratap S, Hulette RB: **Multidrug resistant** ***Acinetobacter baumannii***: a descriptive study in a city hospital. *BMC infectious Diseases.* 2010; 10(1):196. doi: 10.1186/1471-2334-10-196

2. World Health Organization. **Public health importance of antibiotic resistance**. http://www.who.int/drugresistance/AMR_Importance/en/. Accessed 21 Mar 2017.

3. Mandape SN, Marshall DR, Dent LL, Pratap S: **Draft genome sequence of multidrug-resistant** ***Acinetobacter baumannii*** **strain MMC4, isolated from a patient in Tennessee**. *Genome Announc*. 2014; 2(1):e00051-14. doi: 10.1128/genomeA.00051-14.

## P2 Levels of education attainment and income rates to suicide rates in USA: A comprehensive analysis of CDC mortality and US Census data

### Jacob Jones^1^, Toni Brandt^1,3^, Gilberto Diaz^1^, Qingguo Wang^1^, Todd Gary^1,2^, Ashwini Yenamandra^1,3^

#### ^1^College of Computing and Technology, Lipscomb University, 1 University Park Drive, Nashville, TN 37204, USA; ^2^Office of Research, Middle Tennessee State University, 1301 East Main St., Murfreesboro, TN 37132, USA; ^3^Vanderbilt University Medical Center, Nashville, TN 37232, USA

##### **Correspondence:** Ashwini Yenamandra (ashwini.yenamandra@vanderbilt.edu)

**Background**

The Center for Disease Control (CDC) [1] and Tennessee Suicide Prevention Network (TSPN) report consistent findings in an increase of suicide related deaths at the national and state level. Collaborative efforts with the TSPN were initiated by the authors to contribute actionable analysis for significant variables in respect to suicide.

**Materials and methods**

CDC mortality data from 2003 through 2013 was extracted, transformed, loaded and analyzed utilizing Python, R Scripting, RStudio and Tableau. During this comprehensive analysis the CDC mortality data was subsetted into a data frame of 17 variables from the original 75 variables that indicated the most statistical significance as a function of the respective suicide ICD 10 codes.

**Results**

Education attainment levels of 12th grade emerged as one of the most statistically significant variables that contributed to all manner of deaths; this observation is consistent with initial observations of the 2013 CDC mortality data, as it pertained to suicide, analyzed in previous studies [2]. Based on this unique finding of education emerging as a strong and consistent variable in the comprehensive analysis of CDC data over a 11 year period, the authors hypothesized education attainment level segments are the most significant demographic predictor variable of suicide and a systematic approach to targeting continuing educational opportunities to patients with low education attainment levels paired with other high risk segments including but not limited to age, race, ethnicity, marital status and gender [3]. The 2010 census data was used in a hypothesis test to validate the significance of education attainment levels as a significant variable in respect to all manners of death and suicide. A comparison of the distribution between the reported deceased education levels from the CDC data and the education attainment levels of the reported living population from the census data lead the authors to reject their null hypothesis that the distributions of education attainment levels would be consistent between the reported deceased and living populations. The alternative hypothesis was accepted that the distribution of education attainment levels are significantly different among the deceased and living populations. Additional research of the significance of the education attainment level is required to explore the relationship between education attainment levels and the manner of death of the deceased population reported in the CDC data.

References

1. Centers for Disease Control and Prevention. Suicide: Consequences. 2015. http://www.cdc.gov/violenceprevention/suicide/consequences.html. Accessed 10 Jan 2017.

2. Brandt T, Diaz G, Jones J, Gary T, Yenamandra A. **A data science approach to identify previously unknown indicators that could lead to the prevention of suicide in USA**. *Int Clin Pathol J*. 2016; 2(4): 00047. doi: 10.15406/icpjl.2016.02.00047.

3. Diaz G, Jones J, Brandt T, Gary T, Yenamandra A. **Translating Data into Discovery: Analysis of 10 Years of CDC Data of Mortality Indicates Level of Attainment of Education as a Suicide Risk Factor in USA** *Soc Behav Res Pract Open J*. 2017; 2(1) doi: 10.17140/SBRPOJ-2-107.

## P3 Pilot investigation of the competition between commensal and pathogenic staphylococci within the human nasal microbiome

### Marina Z Ghattas^1^, Marwa Elrakaiby^1^, Ramy K Aziz^1^, Hamdallah H Zedan^1^

#### ^1^Department of Microbiology and Immunology, Faculty of Pharmacy, Cairo University, Cairo, Egypt

##### **Correspondence:** Ramy K Aziz (ramy.aziz@gmail.com)

**Background**

The human body is inhabited by trillions of microbes that constitute the microbiome of each individual [1]. The composition of these microbial communities differs from one individual to another depending on gender, diet, genetics, geographical location, etc [2]. Such interindividual diversity affects human health, disease, and even response to therapy [3, 4], and microbiome imbalance has been linked to dysbiosis and disease [1]. Moreover, the human microbiota is a major determinant of the human innate immunity as resident microbes help preventing colonization by external pathogens. A typical example is how the nasal microbiota can protect the host against carriage of multi-resistant *Staphylococcus aureus*. Recently, an inverse correlation was discovered between nasal colonization by *S. epidermidis* and *S. aureus*; therefore, we launched this pilot study to analyze the relative abundance of nasal staphylococci and to investigate the competition between these two staphylococcal species.

**Methods and results**

Duplicate nasal swabs were collected from 17 healthy volunteers living in Cairo Egypt, including 10 nurses. One swab was used for culture-based analysis while the other was subjected to DNA extraction. The V4 regions of the 16S rRNA gene of five nasal swabs were amplified and sequenced on an Illumina MiSeq platform. The biodiversity of bacterial taxa was analyzed by the QIIME software package, and statistical analysis was performed in QIIME and R. Three out of the five samples were quite close to each other (low UNIFRAC distance), while the other two were rather divergent. All samples were dominated by genera *Staphylococcus*, *Corynebacterium*, and *Propionbacterium*, while the most differentiating genera were *Peptoniphilus* and *Anaerococcus* (Samples 1,2,3); *Dialister* and *Prevotella* (Sample 4); *Bacteroidetes* and *Fecalibacterium* (Sample 5).

To validate the data and sample divergence, we compared the microbiome composition of the selected samples with randomly selected publicly available human microbiome samples from various body sites (oral, skin, nasal, intestinal, and vaginal microbiomes: 60-80 samples per site). Interestingly, no geographical signal was detected as the nasal samples from Egyptian individuals clustered with HMP nasal samples, and were most similar to skin microbiomes and least similar to intestinal microbiomes. Because 16S rRNA gene sequencing is not sensitive enough to resolve species-level variations, we used comparative genomics (MetaRef [5]) to define signature genes for *S. aureus* and *S. epidermidis*, and we used PCR to screen all 17 samples for these two potentially competing organisms.

**Conclusion**

This pilot study confirmed the lack of geographical signal among nasal microbiome samples from Egypt. Two distinct types could be identified, one dominated by *Staphylococcus* sp. & *Corynebacterium* sp., while the other is surprisingly rich in *Bacteroides*. PCR analysis of commensal vs. pathogenic staphylococci showed mutual exclusion except one sample only. Ongoing work will focus on using our primers in the quantification of each species in samples.

References

1. Cho I, Blaser MJ: **The human microbiome: at the interface of health and disease**. *Nat Rev Genet* 2012, **13**(4):260-270.

2. Ursell LK, Clemente JC, Rideout JR, Gevers D, Caporaso JG, Knight R: **The interpersonal and intrapersonal diversity of human-associated microbiota in key body sites**. *J Allergy Clin Immunol* 2012, **129**(5):1204-1208.

3. Rizkallah MR, Saad R, Aziz RK: **The Human Microbiome Project, personalized medicine and the birth of pharmacomicrobiomics**. *Curr Pharmacogenomics Person Med* 2010, **8**(3):182-193.

4. ElRakaiby M, Dutilh BE, Rizkallah MR, Boleij A, Cole JN, Aziz RK: **Pharmacomicrobiomics: the impact of human microbiome variations on systems pharmacology and personalized therapeutics**. *OMICS* 2014, **18**(7):402-414.

5. Huang K, Brady A, Mahurkar A, White O, Gevers D, Huttenhower C, Segata N: **MetaRef: a pan-genomic database for comparative and community microbial genomics**. *Nucleic Acids Res* 2014, **42**(Database issue):D617-624.

## P4 Human vs. Nature: Islands and bridges within the microbiome continuum

### Moamen Elmassry^1^, Marwa ElRakaiby^2^, Ramy K Aziz^2^

#### ^1^Department of Biological Sciences, Texas Tech University, Lubbock, TX 79409 USA; ^2^Department of Microbiology and Immunology, Faculty of Pharmacy, Cairo University, Cairo, Egypt

##### **Correspondence:** Ramy K Aziz (ramy.aziz@gmail.com)

**Background**

The completion of the Human Genome Project led to the recognition that many human phenotypes, including variation in disease susceptibility and drug response, cannot be merely explained by human genetic variations [1]. Thus, the Human Microbiome Project (HMP) was initiated to investigate the contribution of human-associated microbes to human phenotypic variations [2, 3]. Meanwhile, rapid advances and cost reduction of sequencing technologies led to the rapid accumulation of genomic, metagenomic, and 16S rRNA data from different habitats, aiming at exploring the biodiversity of almost every samplable ecosystem on earth [4]. Although most of these data were published and interpreted separately, re-examining them in a holistic way may lead to new insights about microbial ecology. Here, we reanalyzed publicly available 16S microbiome sequences from 1,689 samples, representing 17 ecosystems, five of which are human-associated (the five HMP sites), aiming to examine overlaps and correlations between different environments.

**Materials and methods**

16S rRNA gene sequence data were obtained from public databases (e.g., HMP [5] and Sequence Read Archive) and re-analyzed with QIIME [6] for beta-diversity by the unweighted and weighted UNIFRAC distance metric for taxa composition and relative abundance, respectively. Principal coordinate analysis (PCoA) was used for clustering different samples according to their UNIFRAC distances.

**Results**

**Fig. 2 (abstract P4).**
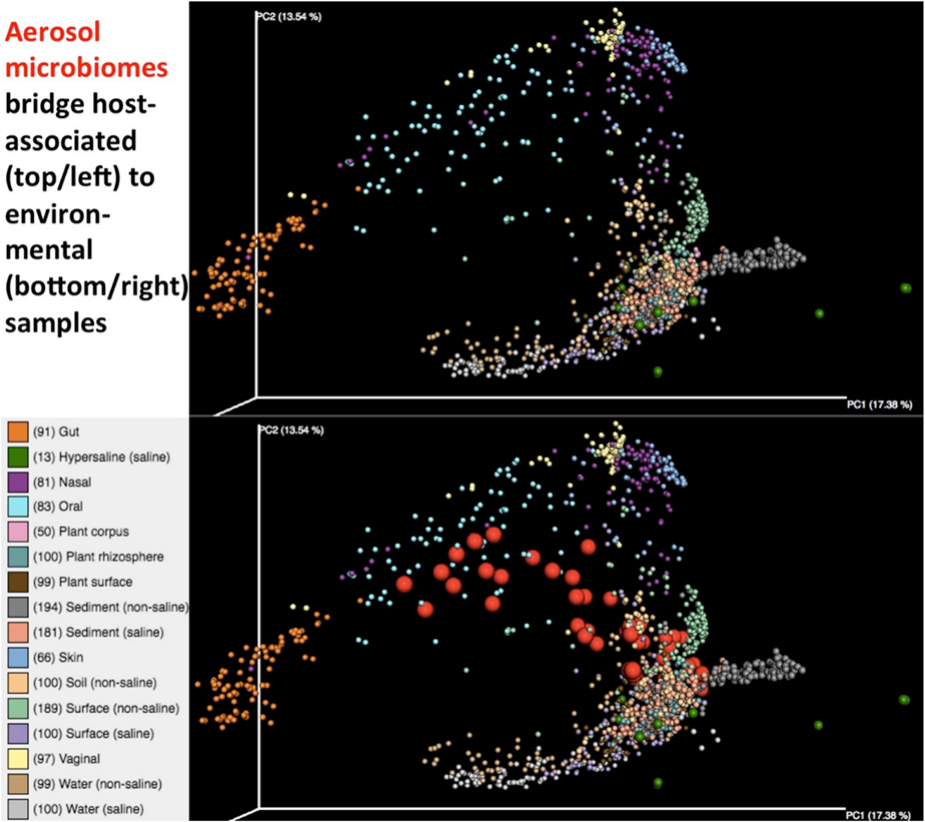
Principal coordinate analysis of weighted UNIFRAC distances demonstrates the various clusters within a global microbiome continuum. Upper and lower panels are identical but for the aerosol microbiomes (lower panel).

We observed a growing microbiome continuum, in which no ecosystem (or human body site) is fully separated from others, but with samples rather spreading to overlap and span multiple clusters. Yet, extreme clusters can be still distinguished, notably human fecal samples, river sediments, and a few microbiomes from hypersaline environments. Among HMP samples, nasal microbiotas were the most diverse, ranging from a majority clustering with skin samples to a few with similarities to oral and gut samples. Overall, human-associated microbiomes were quite distinct (upper and left clusters, Fig. 2) from plant and environmental microbiomes (bottom and right clusters, Fig. 2). Interestingly, aerosol microbiomes (*n* = 46) clearly bridged both worlds (environmental and host-associated samples).

**Conclusion**

In conclusion, we observed a “microbiome continuum” with two large separate clusters (human associated and environmental) bridged by aerosol microbiomes. We expect that the accumulation of future samples may continue to fill the gaps in this continuum.

**References**

1. Rizkallah MR, Saad R, Aziz RK: **The Human Microbiome Project, personalized medicine and the birth of pharmacomicrobiomics**. *Curr Pharmacogenomics Person Med* 2010, **8**(3):182-193.

2. Human Microbiome Project Consortium: **A framework for human microbiome research**. *Nature* 2012, **486**(7402):215-221.

3. Turnbaugh PJ, Ley RE, Hamady M, Fraser-Liggett CM, Knight R, Gordon JI: **The Human Microbiome Project**. *Nature* 2007, **449**(7164):804-810.

4. Gilbert JA, Meyer F, Antonopoulos D, Balaji P, Brown CT, Desai N, Eisen JA, Evers D, Field D, Feng W *et al*: **Meeting report: the terabase metagenomics workshop and the vision of an Earth microbiome project**. *Stand Genomic Sci* 2010, **3**(3):243-248.

5. **The Human Microbiome Project** [http://hmpdacc.org/].

6. Caporaso JG, Kuczynski J, Stombaugh J, Bittinger K, Bushman FD, Costello EK, Fierer N, Peña AG, Goodrich JK, Gordon JI *et al*: **QIIME allows analysis of high-throughput community sequencing data**. *Nat Methods* 2010, **7**(5):335-336.

## P5 Role of the human eye microbiome in development of conjunctivitis

### Mariam Lotfy^1^, Moamen Elmassry^2^, Jarrad Marcel^3^, Rania A Khattab^4^, Maha M Abdelfattah^5^, Jack A Gilbert^6,7^, Ramy K Aziz^4^

#### ^1^Clinical Operations Department, Ray-Clinical Research Organization, Giza, Egypt; ^2^Department of Biological Sciences, Texas Tech University, Lubbock, TX 79409 USA; ^3^Genomic and Systems Biology, Bioscience Division, Argonne National Laboratory, Argonne, IL 60439 USA; ^4^Department of Microbiology and Immunology, Faculty of Pharmacy, Cairo University, Cairo, Egypt; ^5^Research Institute of Ophthalmology, Giza, Egypt; ^6^Department of Surgery, University of Chicago, Chicago, IL 60637, USA^; 7^Microbial Ecology Group, Argonne National Laboratory, Argonne, IL 60439 USA

##### **Correspondence:** Ramy K Aziz (ramy.aziz@gmail.com)

**Background**

The Human Microbiome Project (HMP) was launched to explore human-associated microbes and their impact on human health, disease, and immunity status. The human microbiota at different anatomic sites (mouth, nose, skin, colon, and vagina) is being extensively studied; yet, studies on the eye microbiome are still lagging behind, possibly because the human eye was once considered free of resident microbiota and because of the scarcity of cultured bacteria isolated from the eye [1-3]. The human eye, being in contact with the environment, is continuously exposed to microbes and viruses, but the balance between resident and transient microbes is not fully understood. Here, we use conjunctivitis as a model to understand the potential role of the eye microbiota in health and disease.

**Materials and methods**

We collected duplicate sets of 128 conjunctival swabs, from 54 patients and ten healthy controls. All patients had only one infected eye, and were free of visible viral or fungal infection. Importantly, to minimize inter-individual variations, we compared infected and uninfected eyes of each patient, and we used healthy controls to estimate the natural variability between two eyes. One swab, from each eye, was used for culture-based analysis and the other was processed for DNA extraction and 16S amplicon analysis by high-throughput sequencing (MiSeq, Illumina, USA).

**Results**

**Fig. 3 (abstract P5).**
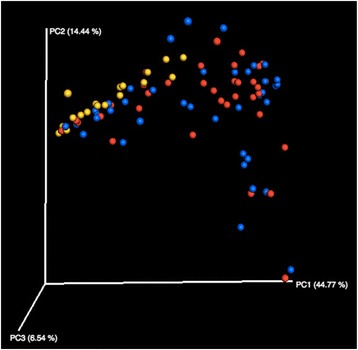
Principal coordinate analysis of weighted UNIFRAC distances demonstrate the absence of particular signature for conjunctivitis.

Sixty-four samples were culture positive. Most commonly isolated bacteria were *Staphylococcus aureus*, *S. epidermidis* and *Acinetobacter*. Antimicrobial susceptibility profiles were mostly similar in both eyes. The microbiome composition of 102 samples (from 51 subjects) was analyzed and compared. Many bacterial taxa were determined that were undetected by culture-based techniques, e.g., *Moraxella, Micrococcaceae* and *Corynebacteria*. When compared to different HMP samples (from HMP and SRA databases), eye microbiome samples clustered together, but were quite close to the skin and nose microbiomes. Beta diversity between subjects was higher than the diversity between eyes of the same subjects (Fig. 3), and no significant differences were seen between the two groups. A few bacterial taxa were significantly more abundant in the control group, e.g., *Pseudomonadaceae*, and *Enterobacteriaceae*. Yet, the biological significance of these statistical differences is not certain because of the small proportion of these taxa. Inter-eye variability was more or less the same among patient and control samples, and interinidividual differences remained the most prominent.

**Conclusion**

In conclusion, our findings suggest that bacteria conjunctivitis is most likely caused by resident taxa of the eye, with no outside pathogen(s) singled out as causative agent(s).

References

1. Aoki R, Fukuda K, Ogawa M, Ikeno T, Kondo H, Tawara A, Taniguchi H: **Identification of causative pathogens in eyes with bacterial conjunctivitis by bacterial cell count and microbiota analysis**. *Ophthalmology*. 2013; 120(4): 668-676. doi: 10.1016/j.ophtha.2012.10.001.

2. Schabereiter-Gurtner C, Maca S, Rolleke S, Nigl K, Lukas J, Hirschl A, Lubitz W, Barisani-Asenbauer T: **16S rDNA-based identification of bacteria from conjunctival swabs by PCR and DGGE fingerprinting.** *Investigative ophthalmology & visual science*. 2001; 42(6):1164-1171.

3. Willcox MD: **Characterization of the normal microbiota of the ocular surface**. *Exp Eye Res*. 2013; 117:99-105. doi: 10.1016/j.exer.2013.06.003.

## P6 Identification of reference genes for quantitative RT-PCR analysis of miRNAs in equine serum

### Pouya Dini^1,2^, Shavahn C Loux^1^, Kirsten E Scoggin^1^, Alejandro Esteller-Vico^1^, Edward L Squires^1^, Mats HT Troedsson^1^, Peter Daels^2^, Barry A Ball^1^

#### ^1^Department of Veterinary Science, University of Kentucky, Lexington, KY 40536, USA; ^2^Department of Reproduction, Faculty of Veterinary Medicine, University of Gent, Merelbeke, Belgium

##### **Correspondence:** Barry A Ball (b.a.ball@uky.edu)

**Background**

MicroRNAs (miRNAs) expression patterns are commonly determined by RT-qPCR; however, the identification of tissue-specific and species-specific reference miRNA is a prerequisite for miRNA expression analysis. The aim of the current study was to identify reference genes for normalization of miRNA expression patterns in serum throughout equine pregnancy.

**Materials and methods**

A list of twenty potential reference genes was initially chosen based on a previously generated miRNA sequencing dataset by NormFinder software (v 0.953). Expression stability of these miRNAs was evaluated in the serum samples collected from different stages of equine pregnancy (4, 6 and 10 month) and post-partum, as well as in the serum of geldings and diestrous mares. Of these, we identified the most stable miRNAs with geNorm (SLqPCR package, Version 1.0.0) and NormFinder. The correlation between the “stability value” generated by NormFinder and the “M value” generated by geNorm was calculated by Pearson correlation coefficient. Spearman’s rank-order correlation coefficient was calculated to assess the correlation between miRNAs ranking with geNorm and NormFinder.

**Results**

NormFinder and geNorm consistently identified eca-miR-21-5p, eca-let-7a-5p and eca-miR-10a-5p as the three most stable reference genes for the normalization of serum miRNAs. Spearman correlation demonstrated a high correlation between the rankings by geNorm and NormFinder, (*rho* = 0.97, *p-value < 0.01*). Similar results were obtained using Pearson correlation coefficient between geNorm average expression stability value “M” and NormFinder “Stability value”(*r* = 0.967 with *p-value* < 0.01). These findings provide three reliable reference miRNAs, eca-miR-21-5p, eca-let-7a-5p and eca-miR-10a-5p, for the evaluation of equine miRNAs expression patterns in serum during equine pregnancy.

**Key words**: equine; microRNA; pregnancy; serum

**Acknowledgements**

This work was supported by the Kentucky Thoroughbred Association/Kentucky Thoroughbred Breeders and Owners, the Albert G. Clay Endowment and the Paul Mellon Postdoctoral fellowships at the University of Kentucky and the Special Research Fund (BOF) at the University of Gent.

## P7 Analysis in modern horses for non-caballine introgression

### Kalpani De Silva^1^, Ernest Bailey^2^, Joel C Stephens^3^, Theodore S Kalbfleisch^4^

#### ^1^Interdisciplinary Studies Program: Specialization in Bioinformatics, University of Louisville, Louisville, KY 40292, USA; Department of Veterinary Science, University of Kentucky, Lexington, KY 40506, USA; ^3^Genomics GPS, Guilford, CT 06437, USA; ^4^Department of Biochemistry and Molecular Genetics, School of Medicine, University of Louisville, Louisville, KY 40292, USA

##### **Correspondence:** Kalpani De Silva (kandaudamalinika.desilva@louisville.edu)

**Background**

Horse breeds have undergone many outcrossing events throughout time. In a recent study, a region of introgression event was identified which is estimated by an evolutionary clock to have occurred about 500,000 years ago. Now we are searching for other events of introgression which might explain adaptive introgression in modern horses.

**Materials and methods**

Our study consists of 6 animals including three horses as well as three non-caballine equids, a kiang, zebra, and a Somali ass. Because our search is for events that happened so long ago, the haplotype blocks for which we are searching are likely to be much smaller than those that have been reported in humans such as the Human x Denisovans or Human x Neanderthal necessitating a different search strategy. Here we present an algorithm and preliminary results of our search for introgression of non-caballines in modern horses.

## P8 Effects of ethanol and lipopolysaccharide on the renal cortex proteome and transcriptome

### Christine E Dolin^1^, Lauren G Poole^1^, Daniel W Wilkey^2^, Eric C Rouchka^3^, Gavin E Arteel^1^, Michelle T Barati^2^, Michael L Merchant^1,2^

#### ^1^Department of Pharmacology & Toxicology, University of Louisville, Louisville, KY 40202, USA; ^2^Department of Medicine, University of Louisville, Louisville, KY 40202, USA; ^3^Department of Computer Engineering & Computer Science, University of Louisville, Louisville, KY 40202, USA

##### **Correspondence:** Christine E Dolin (cedoli01@louisville.edu)

**Background**

The kidney is known to be damaged in end-stage liver disease caused by ethanol (EtOH) consumption (i.e. hepato-renal syndrome) [1]. However, the direct effects of EtOH on the kidney are unclear. Some studies suggest that EtOH damages the kidney (i.e. oxidative stress) [2,3]. These studies have largely been limited to hypothesis-driven approaches based on established mechanisms in other target organs; a discovery-based approach has not yet been applied. Additionally, the effects of EtOH on the kidney’s response to secondary hits are unknown. We hypothesized that moderate EtOH consumption alters renal responses to acute lipopolysaccharide (LPS) exposure. This was investigated using a discovery-based proteomic and transcriptomic approach.

**Materials and methods**

Mice were pair fed EtOH-containing diet for 6 weeks and/or injected i.p. with LPS 4 h prior to sacrifice. Kidney cortex sections were isolated and snap frozen. Comparative Omics studies used (a) two-dimensional liquid chromatography-mass spectrometric analysis with an Orbitrap Elite and TMT labeling reagents and (b) mirVana™ isolation of total RNA, library preparation using the TruSeq Stranded Total RNA LT Sample Prep Kit-Set A with Ribo-Zero Gold and data collection using Illumina NextSeq 500/550 with the RNASeq protocol. Proteomic data were filtered by Benjamini-Hochberg (BH) corrected ANOVA *p*-value <0.05 and fold change (FC) ≥ 1.2. Transcriptomic data were filtered by q-value <0.05 and FC ≥ 2. Ingenuity Pathways Analysis (IPA) was used to identify pathways changed by EtOH and/or LPS. Individual protein changes were validated with immunoblot.

**Results**

EtOH and/or LPS exposure significantly changed expression of 853 of 47,719 transcripts. EtOH and/or LPS also impacted the proteome, significantly changing the abundance of 170 of 1863 proteins. The majority of these effects were LPS-derived. EtOH decreased the abundance of peroxisomal proteins, but this was not caused by a decrease in transcription; EtOH did not decrease expression of peroxisomal transcripts. Interestingly, LPS attenuated the decrease in peroxisomal protein abundance caused by EtOH. IPA results show that EtOH exposure downregulated the Nrf2 pathway, and EtOH preexposure attenuated LPS effects on LXR/RXR and Nrf2-mediated pathways. Western blot and immunohistochemistry of Nrf2 target proteins validated proteomic findings.

**Conclusions**

This study revealed new changes caused by EtOH and/or LPS in renal proteins, transcripts, and pathways. These changes provide insight into mechanisms by which EtOH affects the kidney and alters response to a second pathologic stimulus.

**Acknowledgements**

We would like to acknowledge the contributions of the UofL Department of Pharmacology & Toxicology, the UofL Alcohol Research Center, the UofL Proteomics Laboratories, the UofL Genomics Core Facility, the KBRIN Bioinformatics Core, and the division of Nephrology & Hypertension, Department of Medicine. Grant support provided by NIH grants R01AA021978, P50AA024337, R01DK091584, P20GM103436 and P20GM106396.

References

1. Tsien C, Wong F: **The impact of acute kidney injury in cirrhosis: does definition matter?** *Gut.* 2013; 62:1091-2. doi: 10.1136/gutjnl-2013-304576.

2. Harris PS, Roy SR, Coughlan C, Orlicky DJ, Liang Y, Shearn CT, Roede JR, Fritz KS: **Chronic ethanol consumption induces mitochondrial protein acetylation and oxidative stress in the kidney.** *Redox Biol.* 2015; 6:33-40. doi: 10.1016/j.redox.2015.06.021.

3. Latchoumycandane C, Nagy LE, McIntyre TM: **Chronic Ethanol Ingestion Induces Oxidative Kidney Injury through Taurine-inhibitable Inflammation.** *Free Radic Biol Med.* 2014; **69**:403-16. doi: 10.1016/j.freeradbiomed.2014.01.001.

## S1 Successes and challenges of metabolic reprogramming elucidation in cancers directly from human subjects via Stable Isotope Resolved Metabolomics (SIRM)

### Richard M Higashi^1,2,3^, Teresa W-M Fan^1,2,3^, Hunter Moseley^1,2,3^, Andrew N Lane^1,2,3^

#### ^1^Center for Environmental and Systems Biochemistry, University of Kentucky, Lexington, KY 40536, USA; ^2^Department of Toxicology and Cancer Biology, University of Kentucky, Lexington, KY 40536, USA; ^3^Markey Cancer Center, University of Kentucky, Lexington, KY 40536, USA

##### **Correspondence:** Richard M Higashi (rick.higashi@uky.edu)

**Background**

Diseases such as cancers depend on enhanced nutrient utilization and metabolic reprogramming, the deciphering for which metabolomics at first appears to be well-suited. But each metabolite is used in numerous roles, so that even with excellent metabolite analytical data, the INFORMATION signal-to-noise of each metabolite is less than unity due to participation in multiple pathways. What is needed is to track the provenance of metabolite **sub**structures that reveals the coupled metabolic reactions in a network. Stable isotope resolved metabolomics (SIRM) does just that, to distinguish pathways and functions among otherwise chemically identical metabolites.

**Materials and methods**

SIRM achieves functional proteomics via in situ enzyme assays, revealing up/down regulation of pathways in metabolic reprogramming. This is accomplished by incubating cell cultures with e.g. [U-^13^C]-glucose or [U-^13^C,^15^N]-glutamine [1], infusing human lung cancer patients with [U-^13^C]-glucose and resecting their tumor and non-tumor tissues [2,3], and incubation experiments of their resected tissue slices with [U-^13^C]-glucose or [U-^13^C,^15^N]-glutamine [3]. Some experiments simultaneously incubate the ^13^C and ^15^N and/or ^2^H. Analyses of polar and non-polar metabolites are by multidimensional, multinuclear NMR (e.g. HSQC) combined with ultra-high resolution mass spectrometry; the latter requiring resolving power >400,000 to analyze multiple elemental isotopologues [4].

**Results**

Glucose vs glutamine nutrient sources of carbon in cancers has revealed key aspects of C, N resource allocation, including the up regulation of anapleurosis via pyruvate carboxylase in relation to glucose vs glutamine utilization [1]. In fact, we initially elucidated this pathway in lung cancers resected from ^13^C glucose infused human subjects [2], and further detailed the metabolic reprogramming in humans, tissue slices, and cell cultures [3]. This demonstrates the unique ability of SIRM for untargeted discovery of metabolic networks directly in humans for maximal relevance, comparing the identical experiments in animal and cell models for unprecedented congruence of results. Among the key findings from simultaneous ^13^C, ^2^H experimentation is the mapping of preferred pools of metabolites for e.g. nucleotide vs protein synthesis; these apparent “channeling” pathways are previously unknown, and significant enough to impede or misdirect metabolic modeling.

**Discussion**

One of the greatest challenges is the metabolic landscape revealed by SIRM: that of shared/isolated metabolic pools corresponding to previously unknown dynamic compartmentation. This crucial area is poorly addressed by all of the omics, in part due to lack of extremely detailed, dynamic metabolic phenotype information, which SIRM can help to remedy.

**Acknowledgements**

This work was supported in part by National Science Foundation EPSCoR infrastructure grants EPS-0447479 and EPS-0132295; NIH National Center for Research Resources (NCRR) grants 5P20RR018733, 1R01CA118434-01A2, 1RO1CA101199-01, 3R01CA118434- 02S1, and 1R01ES022191-01, P01CA163223- 01A1, and 1U24DK097215-01A1; the University of Louisville CTSPGP/ARRA grant 20044; the Kentucky Lung Cancer Research Program grants OGMB090354B1 and OGMB101380; the Robert W. Rounsavall Jr. Family Foundation; the Kentucky Challenge for Excellence and Drive Cancer Out Campaign. We thank the co-authors in the publications below, and Melissa Hall and Bridgett Curry for clinical support.

References

1. Le A, Lane AN, Hamaker M, Bose S, Gouw A, Barbi J, Tsukamoto T, Rojas CJ, Slusher BS, Zhang H, Zimmerman LJ, Liebler DC, Slebos RJC, Lorkiewicz PK, Higashi RM, Fan TW-M., Dang CV: **Glucose-independent glutamine metabolism via TCA cycling for proliferation and survival in B-cells.** *Cell Metabolism.* 2012*;* 15:110-121. doi: 10.1016/j.cmet.2011.12.009.

2. Fan TW, Lane AN, Higashi RM, Farag MA, Gao H, Bousamra M, Miller DM: **Altered regulation of metabolic pathways in human lung cancer discerned by** ^**13**^ **C stable isotope-resolved metabolomics (SIRM).** *Mol Cancer.* 2009; 8:41. doi: 10.1186/1476-4598-8-41.

3. Sellers K, Fox M, Bousamra II M, Slone S, Higashi R, Miller D, Wang Y, Yan J, Yuneva M, Deshpande R, Lane A, Fan T: **Pyruvate carboxylase is critical for non–small-cell lung cancer proliferation**. *J Clin Invest.* 2015; 125(2):687–698. doi:10.1172/JCI72873.

4. Lorkiewicz P, Higashi RM, Lane AN, Fan TW-M: **High information throughput analysis of nucleotides and their isotopically enriched isotopologues by direct-infusion FTICR-MS.** *Metabolomics*. 2012; 8:930-939. doi: 10.1007/s11306-011-0388-y.

