# Correction to: Proceedings of the 16th Annual UT-KBRIN Bioinformatics Summit 2017: proceedings

**DOI:** 10.1186/s12919-017-0096-3

**Published:** 2017-11-15

**Authors:** Eric Rouchka

**Affiliations:** Department of Computer Engineering & Computer Science, University of Louisville, Louisville, KY 40202 USA

## Correction

Following the publication of the original supplement article [1], it was brought to our attention that the supplement title unfortunately contains an error: “Proceedings of the 16th Annual UT-KBRIN Bioinformatics Summit 2016: proceedings” should instead read “Proceedings of the 16th Annual UT-KBRIN Bioinformatics Summit 2017: proceedings”.

The correct title has been included in this correction.
